# The Journey of Prepectoral Breast Reconstruction through Time

**DOI:** 10.29252/wjps.10.2.3

**Published:** 2021-05

**Authors:** Sharat Chopra, Zaid Al-Ishaq, Raghavan Vidya

**Affiliations:** 1 Breast Centre, University Hospital Llandough, Penlan Road, Cardiff, Wales, United Kingdom.; 2 The Royal Wolverhampton N.H.S. Trust, Wolverhampton Road, Wolverh-ampton United Kingdom.

**Keywords:** Prepectoral reconstruction, Breast, Acellular dermal matrix, Synthetic mesh, Complications, Radiotherapy

## Abstract

**BACKGROUND:**

The facet of breast reconstruction has evolved from complex surgery to simple implant-based breast reconstruction. Minimal invasive surgery or Prepectoral breast reconstruction has revolutionised the surgical treatment for breast cancer and became a reality due to advances in meshes and implants. In this review, we have looked at the journey of Prepectoral implant beast reconstruction through time.

**METHODS:**

We conducted a literature review on pre-pectoral breast reconstruction, emphasising types of cover, its outcomes, complications, and the effect of postmastectomy radiotherapy.

**RESULTS:**

Prepectoral breast reconstruction had advanced with time and appears to be a safe and effective method of breast reconstruction and is associated with minimal morbidity whilst providing adequate cosmesis. Radiotherapy seems to be well tolerated with early favourable results. The Implant loss rates in the Acellular Dermal Matrix (ADM) to be around 5%-6% and rippling appear to be a common adverse effect of this technique ranging from 0%-35% in various studies.

**CONCLUSION:**

Prepectoral implant-based breast reconstruction has emerged as a successful method of breast reconstruction.

## Introduction

Around one million new breast cancer cases are diagnosed worldwide each year, with nearly 55,000 new breast cancers diagnosed alone in the United Kingdom^1^. The surgical management of breast cancers has changed significantly over the past decade with the availability of various breast reconstructive techniques, especially implant-based reconstruction following a mastectomy. Nearly four out of ten women in the United Kingdom now undergo a therapeutic mastectomy as their choice of the procedure either due to oncological reasons or personal preference^1^. With further advances, development of synthetic (Vicryl^®^, Titanium or TIGR^®^Mesh) and biological material, i.e., Acellular Dermal Matrix (ADM) for implant-based reconstructions, the traditional post pectoral implant-based reconstruction has given way to less traumatic and minimally invasive prepectoral breast reconstruction. 

This descriptive review aimed to look at the journey of prepectoral implant-based reconstructions with time looking into patient selection, anatomy and focusing on its outcomes.

***Time trends and Breast Reconstruction***

Breast reconstruction has come far since 1895, when a fist-sized fatty tissue (lipoma) was transplanted from the patient’s lumbar region to the chest wall following a mastectomy^2^. Several years later, in 1963, the use of silicone prosthesis in implant-based reconstruction was introduced as a delayed procedure, following mastectomy^3^. Thereafter, first immediate breast reconstruction was reported using a silicone implant in a mastectomy patient^3^. The implant breast reconstruction technique in the pre-pectoral subcutaneous plane was initially rejected during the 1980s due to its high complication rates. With higher capsular contracture rates and poor aesthetic outcomes, this technique was temporarily abandoned. Breast reconstruction was achieved with placement of the implant behind the muscle in a total sub-muscular pocket to cover the implant. With significant technical limitations to the sub-muscular implant over the years, such as the inability to achieve an adequate subpectoral pocket to accommodate a fixed volume implant and increased postoperative pain and morbidity to the muscle led to the use of two-staged reconstruction using an expander. In this, the prosthesis was inflated slowly over time to stretch Pectoralis Major and Serratus Anterior muscle and then substituted later with a fixed volume implant. Following complications, the total submuscular technique was replaced by a new dual-plane method or partial coverage technique. 

**Figure 1:**
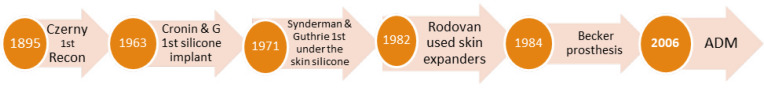
Evolution of Breast Reconstruction

The technique involves partial implant cover with pectoralis major muscle superiorly and inferiorly by the mastectomy skin flap. This technique allows for an improved lower pole expansion but led to excessive “bottoming out” of the breast’s lower stretchy part. A newer modified dual plane technique was practised following the induction of Acellular dermal matrix(ADM) or mesh around 2006-2007. The ADM is a fold of dermis, sutured as a sling for the breast’s lower pole, thereby attaching to the inferior edge of the detached pectoralis major muscle superiorly and inferiorly to the inframammary fold. The ADM covered for the implant both laterally and inferiorly, thereby reducing the incidence of implant migration. However, it was disadvantaged by the potential impairment to shoulder dysfunction and animation deformity due to muscle detachment. Hence, a more novel pre-pectoral approach with or without ADM or synthetic mesh re-emerged avoiding such complications (Figure 1). 

 A wide variety of biological and synthetic meshes are available nowadays. A biological mesh usually referred as an acellular dermal matrix (ADM) is a fold of dermis, which is pre-sterilised for usage. It is obtained from either cadaveric human (Alloderm®), porcine (Strattice®, Permacol®, Braxon®)or bovine (SurgiMend®, Veritas®)source. Biological fold allows rapid host revascularisation and cell re-growth, which facilitates an excellent outcome.

As an alternative to ADMs, Synthetic matrices/meshes can sometimes be used in breast reconstruction. These can either be absorbable (Vicryl^®^), long-term absorbable (TIGR^®^), or a nonabsorbable titanium-coated polypropylene mesh (TiLOOP^®^).

***Technique***

The prepectoral space is a space between the breast skin envelope and chest wall muscles^4^. It is vital to understand this plane to preserve the vascularity of mastectomy flaps for a good reconstruction^4^^,^^5^. An appropriate assessment of the skin flap thickness along-with the understanding of the blood supply of the skin flaps^6^ is imperative to the operation’s success. Pre-pectoral breast reconstruction involves creating a new breast constructed by covering the implant with the mesh/ ADM following a mastectomy and subsequently attaching it over the chest wall, thereby keeping the pectoralis major and serratus anterior undisturbed. The breast remains in its anatomical plane, minimises morbidity, achieves the desired cosmesis, and maintains shoulder functionality.

Prepectoral implant breast reconstruction is a suitable technique following both skin-sparing mastectomy and nipple-sparing mastectomy, either as one stage or a two-stage procedure^7^ depending on the quality of breast skin and breast volume. 

Following mastectomy, two types of implant coverage techniques are used with ADM or synthetic mesh in prepectoral breast reconstructions. First, the complete or total wrap technique uses either full pre-formed ADM(Braxon^®^), for the implant or covering the entire implant with a single (or two) 

ADM sheets. By leaving a small cuff of ADM on the sides, sutures are then placed to secure the ADM to the inframammary fold (I.M.F.) and surroundings. The second technique, the anterior wrap, uses ADM to partially cover the implant (anteriorly), thereby forming a breast pocket. ADM is then sutured superiorly (to the pectoralis muscle) and inferiorly, after placing the implant in the pocket created. The ADM is usually fenestrated, which allows the drainage of fluids through and helps in tissue integration over time. Post-operatively, one or two drains are placed in the reconstruction to drain fluids or collections. The significant advantages of the prepectoral technique are reduced muscle displacement/dissection, less trauma, reduced postoperative discomfort and pain, with avoidance of animation deformity avoidance.

***Literature Review***

We looked at various prepectoral breast reconstruction studies conducted since 2014. We compared studies by type of techniques (synthetic mesh or ADM); partial or complete wrap technique and summarised various complications in each study- both major and minor such as implant loss rates, seroma rates, surgical site infection rates and cosmesis (Table 1).

Eligible studies reporting on outcomes, complications, types of reconstruction were considered for this review. All studies looked for comparability, interest and outcomes within prepectoral breast reconstr-uction technique either comparing with sub-pectoral technique or on its own. Outcomes included both major and minor complications for both total wrap and partial wrap technique and were analyzed below. 

***Implant loss***

The rate of implant loss was recorded in the majority of the reviewed studies (22 studies). Interestingly, the implant loss rate decreases with the amount of mesh used to wrap the implant. While in full wrap cohort^10^^,^^13^^,^^14^^,^^16^^,^^19^^,^^21^^,^^24^^,^^26^^,^^28^^,^^32^, the rate ranges from less than 1%-18% , it decreases to 0-8% in the anterior/ partial cover^12^^,^^17^^,^^18^^,^^20^^,^^22^^,^^25^^,^^27^^,^^31^ and it became even 0% in two studies where no mesh was used (total 47 patients)^15^^,^^23^. Of the full wrap studies, Downs et al., in their retrospective review of 45 patients (79 Alloderm or FlexHD assisted prepectoral implant-based breast reconstruction) correlate their implant loss rate of 18% to their learning curve and not objectively measuring tissue perfusion ^10^. In a prospective study on 64 patients (78 ADM assisted (Braxon) prepectoral implant-based breast reconstruction) reported an implant loss rate of 10% which was comparable to NMBRA results and concluded no superiority of prepectoral to subpectoral implant breast reconstruction in this regard^19^. This study agreed with a later study^31^, that reported in their retrospective study of prospectively collected data comparing subpectoral implant-based breast reconstruction with prepectoral ADM assisted implant-based breast reconstruction. When only PMRT groups were considered (31 vs 26 reconstructions respectively), they found no statistically significant implant loss rate between the two groups with 19.3% and 15.4%. The majority of prosthesis removal was due to infection. Their explanation of submuscular implant loss rate being higher than the prepectoral group was related to the patients’ desire in the submuscular group to request implant removal following breast irradiation due to implant migration, capsular contracture, and clinical discomfort. This group elected to have these reconstructions converted to either an autologous option or a new prepectoral plane. Other authors^13^^,^^14^^,^^16^^,^^17^^,^^18^^,^^21^^,^^24^^,^^26^^,^^28^^,^^32^ reported a comparable rate of implant loss ranging between 1%-5%. While in the anterior/partial wrap studies, Payder et al. in their retrospective review of 10 patients, underwent ADM assisted direct-to-implant or 2-stage breast reconstruction in the prepectoral plane^25^. They reported a loss of 5.6% (1 implant loss) with anterior implant coverage using fenestrated ADM. They proposed not to limit this procedure to patients with small or medium breasts or to those who have undergone a nipple-sparing mastectomy. Of the studies where no mesh had been used, authors reported no implant loss following their retrospective review of 26 and 21 patients who underwent direct implant breast reconstruction in the prepectoral plane following a mean follow up period 51.5 and 4 months respectively^15^^,^^23^.

***Seroma***

Nineteen studies reported the rate of seroma formation following a prepectoral implant-based breast reconstruction with a variable rate to depend on the type of implant coverage (full wrap, anterior wrap, and no mesh). The highest rate found in full implant coverage was 24% reported by Jafferbhoy et al. in their prospective study of 64 patients (78 ADM assisted (Braxon) prepectoral implant-based breast reconstruction)^19^. Aspiration was performed in 23% of patients of whom two patients required repeat aspirations. Their finding was attributed to ADM acting as a tissue regeneration layer between the skin and implant, leading to excess seroma formation. To reduce seroma, Payder et al. advocate ADM fenestrations which improve fluid egress. This reduces potential dead space by facilitating ideal effacement of the ADM with the implant, and the mastectomy flaps ^25^. Downs et al., in their retrospective review, reported about 15% (12/79 breasts) of seroma following a mean follow up of 23 months. Their univariate analysis found that patients with high BMI and smoking history have an increased risk of seroma^10^. Five of them were clinically significant, and one required antibiotic while the remaining four were associated with infection and flap necrosis that necessitate explantation. The authors tend to leave the drain bit longer to reduce the risk of seroma. A similar finding reported by Reitsamer et al. in their prospective review of 134 patients (200 nipple-sparing ADM assisted prepectoral breast reconstructions) following a median follow up of 36 months. The seroma had to be punctured once in half of them and >= two times in the rest^32^. Of note, Casella et al. in their prospective review of 63 patients (73 mastectomies) comparing between retropectoral immediate implant-based breast reconstructions and immediate reconstructions in the prepectoral plane using TiLoop® Bra mesh (34 vs 39 reconstructions respectively) reported no seroma over a follow-up period of 12 months^8^. They explained their low complication rate by restrictive selection criteria of the study design and their previous ADM experience. Interestingly, De vita et al. reported no seroma rate in their prospective review of 21 patients (34 nipple-sparing immediate breast reconstruction using polyurethane implants in the prepectoral plane with no mesh) during follow up of 4 months. This finding could be related to integrating polyurethane substance into the adjacent tissue and reducing the dead space where seroma could form^29^.

***Capsular contracture***

Most of the studies presented in this review showed a favourable aesthetic outcome of capsular contracture rates (Table 1). Use of ADM has led to a reduction in capsular contracture with no evidence in the published literature about the superiority of any individual ADM over the other. The reviewed literature in this study showed a capsular contracture rate varies from 0-10%. Sigalove et al. in their retrospective review of 33 patients (55 ADM assisted prepectoral implant-based breast reconstruction) reported no capsular contracture over a follow up of 25.1±6.4 months^16^. It is recognised that the acellular dermal matrix reduces capsular contracture as it diminishes the inflammatory and profibrotic reactions of breast capsule development. This leads to thinner capsules than native breast capsules. Similar findings have been reported^9^^,^^22^. On comparing the outcome of breast reconstruction (prepectoral vs subpectoral) following PMRT, Sinnott et al. found in their retrospective review a comparable rate of capsular contracture between the two groups with a grade 3 or 4 capsular contracture rate of 5.6% for prepectoral reconstruction group following a follow-up of 20.6±15.4 months^24^. It is almost half of the rate reported (10%) in another retrospective review of 45 patients, 79 breasts treated with immediate prepectoral implant reconstruction over a median follow up of 22 months. With majority reported in this study as Baker II. The study also found Smoking was associated with a significantly higher risk of postoperative capsular contracture^10^. In a study assessing the outcomes following prepectoral implant-based breast reconstruction without mesh, Salibian et al. review of 155 patients (250 breasts) with nipple-sparing mastectomy using prepectoral staged expander/implant reconstruction with thick mastectomy skin flaps without acellular dermal matrix, with a follow-up period of 55.5 months reported a capsular contracture rate of 7.6%. Capsular contracture is time-dependent and may occur after 2 to 3 years^15^. Singla et al. their retrospective review of patients who underwent prepectoral implant-based breast reconstruction without mesh reported only 3.8% over a median follow-up of 51.5 months. This finding could be attributed to the use of a textured implant.^23^ This is in line with a later study by De vita et al. who reported no capsular contracture following the use of polyurethane implant in the prepectoral plane without ADM after a follow-up period of 4 months. They have a high-fat grafting rate (19.2%) which could have a protective effect against capsular contracture^29^. As stated earlier the capsular contracture is time-dependent and considering the short follow up period of the above study; further studies are needed with a long follow up period to evaluate the aesthetic outcome of using polyurethane implant in the subcutaneous plane.

***Infection***

There is no consensus in managing mastectomy pocket before siting the implant, the duration of postoperative prophylactic antibiotic and the amount of drainage needed to dictate drain removal. Perhaps, this could explain the variable rate of infection in the reviewed studies. Besides, there is an overall lack of defining surgical-site infection criteria established in many publications and the significant heterogeneity in its definitions in the literature, making comparisons between studies challenging. In the reviewed studies, the infection rate varies between 0%-14%. 

Vidya et al. reported^14^ in their prospective review of 100 patients underwent prepectoral breast reconstruction using the preshaped acellular dermal matrix for complete breast implant coverage, reported no infection in their cohort after a follow-up period of 17.9 +/-3.6 months. In line with this result, others reported no infection in their studies^8^^,^^11^^,^^12^. In most of the studies, the infection was treated conservatively. However, an implant loss was reported due to infection with a rate of 5% (1 patient) and 2.7% (2 patients) respectively.^25^^,^^22^

Elswick et al. in their retrospective review of 54 patients who underwent two-stage prepectoral implant-based breast reconstruction with postmastectomy radiation therapy found the infection rate was higher in the irradiated breast compare to non-irradiated one (18.8% vs 7.7% respectively)^27^. 

In the group of patients who underwent prepectoral implant reconstruction without mesh, De vita et al. reported no infection in their retrospective review of 34 nipple-sparing prepectoral implant reconstruction with polyurethane over four months follow-up period^29^. However, in a similar study by Singla et al. reported an infection rate of 15% (Minor 11.5% and Major 3.8%)^23^. This finding could be related to the latter study’s seroma rate (15.3%) with its tendency to get infected and cause superficial cellulitis. 

***Rippling***

Six studies reported the finding of rippling after prepectoral implant-based reconstruction. This rate varies from 0 -35%. Downs et al. reported in their retrospective study of ADM assisted prepectoral implant-based breast reconstruction, a 35% (28/79 patients) rippling rate. The majority of patients being thin and required fat injection as a day case procedure^10^. An earlier study reported no rippling in their small case series of 13 patients (22 breasts) who underwent similar procedures^9^. Singla et al. reported 19% contour defect in their prepectoral implant-based reconstruction without mesh which required fat injection^23^. They attributed this high-fat injection rate as they did not use any mesh or ADM coverage of the implant, which may theoretically make contour defects more pronounced. However, all cases were managed as a day-case procedure with no subsequent complications. Casella et al., in their prospective review of 250 prepectoral implant-based breast reconstructions using TiLoop Bra mesh and after two years of follow up reported 16.7% rate of rippling for which their patient required lipofilling^30^. Flap thickness and patient weight could influence postoperative visibility of implant rippling as in their series, overweight patients with thicker flaps did not report visible implant rippling^8^. Salibian et al. recommend in thin individuals (body mass index <21 kg/m2) an implant should be larger than the pocket size for the closure of the wound under moderate tension to minimize postoperative rippling^15^.

Jones et al. reported a 12.3% rate of rippling, which they relate to the use of cohesive gel implants with stiffer consistency^22^. Interestingly, in a retrospective review of 21 patients that underwent prepectoral implant-based breast reconstruction with a polyurethane implant, reported no rippling during their mean follow up of 6 months. However, a larger group of patients are required with longer follow-up to have a better evaluation of this procedure^29^. 

***Breast reconstruction and radiotherapy***

Post-mastectomy radiotherapy is indicated in breast cancers to reduce the risk of local recurrence. Radiation therapy causes both immediate and delayed effects on breast and surrounding tissues. Immediate or early radiation effects are erythema, desquamation, hyperpigmentation, oedema and ulceration with nearly 85% of patients showing acute radiation dermatitis^33^. Delayed radiation tissue changes in the breast were telangiectasia, skin dryness, discolouration leading to fibrosis of the skin, ultimately leading to more complications such as higher capsular contracture rates, implant failures and poor cosmesis^34^.

We looked at the available evidence of the effects of radiotherapy and breast reconstruction (Table 2).

In the United Kingdom, nearly 30%-40% of women are not offered Immediate Breast Reconstruction(IBR) because the possibility of Post Mastectomy Radiotherapy Treatment(PMRT) is equivocal or unknown at the time of mastectomy. As a result, breast reconstruction may be delayed until the final pathology is available and the need for radiotherapy is confirmed.

A survey by the Association of Breast Surgery in the United kingdom^35^ ( Mar to Jun 2014) showed that most surgeons (77%) believe the current evidence base for breast reconstruction is insufficient to guide decision-making regarding reconstruction. Nearly 90% will opt for a delayed reconstruction in the face of radiotherapy even though there was no difference in the quality of life and or cosmesis between immediate vs delayed reconstructions.

Earlier studies, review of two-stage, AlloDerm-assisted, prosthetic breast reconstructions from 2004 to 2010 showed capsular contracture rates (grade III/IV) to be significantly higher in the radiation therapy group^36^.

A recent study compared the outcomes of patients who underwent immediate, direct-to-implant, or 2-staged, prepectoral breast reconstruction followed by PMRT with those from patients who did not receive PMRT and have demonstrated that the capsular contracture rates to be comparable in both the radiated and non-irradiated group^16^. Another study conducted a retrospective review of consecutive patients undergoing immediate two-stage prepectoral implant-based breast reconstruction with postmastectomy radiation therapy. Outcomes of irradiated breasts compared with non-irradiated breasts in bilateral cases. On univariate analysis, there were no risk factors associated with any complication, including radiation therapy and surgical-site infection, and showed low rates of capsular contracture of 1.9%, seroma 5.6% and implant extrusion of 1.9%^27^ .

Some authors have reported adverse effects of radiation on breast prosthesis due to fibrosis of the pectoralis muscle. This leads to implant migration noticeably seen in subpectoral reconstructions not in Prepectoral breast reconstruction^7^^,^^31^. They have also recorded lower implant loss rates in the Prepectoral group (15.4%) than the subpectoral group (19%).

A retrospective study was conducted in which they compared the effect of PMRT on patients who had ADM assisted implant-based reconstruction without PMRT (158 patients) versus those who had same reconstruction procedure followed by radiotherapy (28 patients). They found no significant risk of implant loss 1.1% vs 3.6%, rippling of 0.6% vs 0, seroma 8% vs 7.1%, and infection 5.2% vs 0 between the two groups. However, the capsular contracture rate was higher in the PMRT group, 10.7% compared to non-PMRT group 0.6%, which was statistically significant ^37^^,^^38^. 

In comparison, the effect of PMRT in patients who had subpectoral implant-based breast reconstruction versus prepectoral one, contracture rate was significantly higher for the subpectoral patients with PMRT than for the prepectoral patients with PMRT (52.2 vs 16.1%; *P*=0.0018) ^24^. The severity of capsular contracture (Baker grades 3 or 4) was more in the subpectoral cohort that received PMRT. In contrast, Sbitany et al. reported in their prospective study of 31 breasts of subpectoral implant-based reconstruction in comparison with 26 breasts of prepectoral cohort followed by PMRT and found no statistically significant differences in infection, seroma, or explantation rates^31^.

## DISCUSSION

 We looked at various papers on prepectoral breast reconstructions with ADM / synthetic mesh studies in this descriptive review. Overal, 2000 patients with prepectoral reconstruction with ADM /synthetic mesh were included since 2014. We looked at various complications ranges for groups with full wrap technique vs partial wrap technique for implant coverage. We found the overall complications ranged, for both techniques (Table 3).

Our paper noted the pooled analysis of all the studies suggesting that the prepectoral reconstruction technique had some complications. Overall Seroma rates^10^^,^^15^^,^^23^^,^^24^^,^^26^^,^^27^^,^^30^ and the surgical site infection rates ^7^^,^^10^^,^^13^^,^^14^^,^^17^^,^^18^^,^^19^^,^^23^^,^^25^^,^^28^^,^^30^^,^^31^^,^^32^ in the publications were noted to be higher in the ADM group as shown in Table 3. The average follow-up range in studies was between 4-55 months. Comparing all ADM studies, average seroma rates were around 7%-8% in this group while the studies using TiLoop mesh showed no seromas. In comparison, a study showed (used of TIGR^R^ mesh and ADM) seroma rates to be around 14%. This study divided patients based on surgeon’s discretion into either ADM group (56%) or TIGR mesh group (44%) and included patients with postoperative radiotherapy (16%), which could account for these high rates. ^32^

The capsular contracture rates documented in the papers in the prepectoral reconstructions to be around 3%-10%. Upon subgroup analysis, the reported higher rates of capsular contractures in some studies has significantly reduced over time, with the use of ADM. We noted the average capsular contracture rates in partial wrap ADM to be lower(3.6%) than in full wrap ADM use (5.2%) which in comparison to the Tiloop^R^ mesh is around 4%^8^^,^^30^. Surgeons’ experience and knowledge of understanding of prepectoral anatomy has significantly evolved over time^15^^,^^24^^,^^30^^,^^38^.

We also noted major complications such as the implant loss rates^10^^,^^19^^,^^25^^,^^26^^,^^31^^,^^32^ in a range of 0%-18% between two groups with average Implant loss rate to be around 5.2% with the ADM and 3%-3.5% in the synthetic mesh group.

Red breast syndrome associated with the use of ADM noted in nearly 6% cases. Most of this was self-limiting, but some were treated with antibiotics for a week and then observed^39^. 

Pooled averaged rates for complications such as wound infection, hematoma, and skin necrosis from the above studies recorded were around 2%,1%-2% and 3%-4% respectively.

 Several articles published on PubMed dating back in time have reported complications rates to be much higher than the most recent studies. With time, we believe the surgeons’ experiences and techniques performing the prepectoral breast reconstruction have evolved. With a careful patient selection, types of incisions used, and better recording/registry of complications, we can reduce significant postoperative complications. 

In patients with Prepectoral breast reconstruction with ADM’s or synthetic mesh with post-mastectomy radiotherapy, we found studies to be in favour of use of radiotherapy^31^^,^^37^^,^^38^. Prepectoral breast reconstruction group had minimal or comparable complication rates (25% in irradiated vs 23% in the non-irradiated group) compared with the subpectoral group (Table 2).

Similarly, in a study by Sinnott et al. ^24^ showed the rates of capsular contracture were three times higher in subpectoral reconstruction than the prepectoral group who were irradiated, showing perhaps the protective role of ADM in prepectoral reconstruction.

The two published series using neither ADM nor synthetic mesh in prepectoral reconstruction for comparison found series exploring patients’ feasibility and outcomes. These patients underwent immediate breast reconstruction using skin-sparing mastectomy using a vertical inframammary incision. Proper patient selection and skin flap viability are the key to achieving optimal outcomes without using ADM or a mesh in prepectoral breast reconstruction^23^. Similarly, astudy of 155 patients with no mesh in a nipple-sparing mastectomy with prepectoral reconstruction with a follow up of 55.5 months showed higher complication rates. There was poor cosmesis in 19% of patients who needed a second procedure( fat grafting), perhaps suggesting ADM’s protective role in prepectoral breast reconstruction^15^^,^^23^.

**Table 1:** Literature review on studies on prepectoral breast reconstruction

**Year**	**Author**	**Implant loss rate**	**seroma rate**	**capsular contracture**	**Cosmetic Issue** **Rippling**	**S.S.I**	**Type of Mesh: Material used + (Type of cover)**
2014	Casella et al. (n=34)^8^	3	0	n/r	n/r	0	Titanium mesh (full cover)
2015	Reitsamer et al. (n=13)^9^	n/r	n/r	0	0	n/r	Porcine ADM/mesh (full cover)
2016	Downs et al. (n=45)^10^	18	15	10	36	10	Human ADM (full wrapped)
	Bernini et al. (n=34)^11^	3	0	n/r	n/r	0	Titanium mesh (full cover)
	Caputo et al.(n=27)^12^	0	n/r	n/r	n/r	0	Porcine ADM (partial anterior cover)
2017	Vidya et al. (n=51 & n=79)^13^^,^^14^	1-2	7	n/r	n/r	<2	Porcine ADM (full cover)
	Salibian et al.(n=155)^15^	n/r	n/r	8	<4	2-3	None
	Sigalove et al. (n=207)^16^	2	2	n/r	n/r	n/r	Human ADM (full cover)
	Nahabedian et.al. (n=39)^17^^,^^18^	5	5	n/r	n/r	<9	Human ADM (anterior/ partial cover)
	Jafferbhoy et al. (n=64)^19^	10-11	24	n/r	n/r	6-7	Porcine ADM (full wrap)
	Sbitany et al. (n=51)^20^	1-2	4	n/r	n/r	7-8	Human ADM (anterior /partial cover)
	Highton et al. (n=106)^21^	3	3	n/r	n/r	n/r	Porcine ADM (full cover)
	Jones et al. (n=50)^22^	3	13	0	12-13	<6	Human ADM (anterior/ partial cover)
	Singla et al.(n=26)^23^	0	15	<4	19	14	None
2018	Sinnott et al.(n=274)^24^	4	<1	5-6	<1	<3	Human ADM(full cover)
	Payder et.al (n=10)^25^	6	0	0	n/r	<6	Fenestrated ADM (anterior partial cover)
	Chandarana et al. (n=61)^26^	4	<2	<2	n/r	<7	Porcine ADM (full cover)
	Elswick et al.(n=54)^27^	1.5	<6	<2	n/r	14	Porcine ADM (Anterior/partial cover)
	Cattelani et. al (n=86)^28^	<1	n/r	n/r	n/r	1	Porcine ADM (full cover)
2019	De vita et al.(n=21)^29^	0	0	0	n/r	0	Polyurethane coated implants
	Casella et al.(n=187)^30^	n/r	1	<4	3-4	3	Titanium Mesh (fully wrapped)
	Sbitany et al.(n=175)^31^	<8	n/r	n/r	n/r	4	Human ADM (anterior/partial cover)
	Chandarana et al. (n=61)^26^	4.3	<1	n/r	n/r	1-2	Porcine ADM (full cover)
	Reitsamer et.al. (n=134)^32^	3.5	14.5	n/r	3.5	7	Porcine ADM/synthetic mesh (full cover)

Complications (in percentages)

n/r= Not Recorded

SSI= Surgical Site Infection n= Number of patients

**Table 2:** Effects of Radiotherapy and Implant Breast Reconstruction

**Year**	**Study**	**Findings**
2012	*Scott Spear*	AlloDerm-assisted prosthetic breast reconstruction, irradiation showed higher rates of clinically significant capsular contracture^36^.
2017	*Sigalove S, Maxwell GP*	Retrospective data n=93 patients found no difference in adverse effect outcomes with PMRT and Prepectoral Reconstruction^16^
2018	*Elswick SM, Harless CA*	Retrospective data n=52 patients found no difference in adverse effect outcomes with PMRT and Prepectoral Reconstruction.^27^
	*Sinnott CJ, Persing S.M.C.*	Sub-pectoral breast reconstruction with PMRT had a greater rate of capsular contracture than in pre-pectoral reconstruction^24^
2019	*Sbitany et al.*	This study illustrates that, in all patients, regardless of radiation therapy status, the infection rate in prepectoral patients is slightly higher than in the submuscular cohort. However, in the setting of postmastectomy radiation therapy, there is no statistically significant variation in infection rates between the two cohorts^31^.
	*ChandersekaranS, Apte A*	The study looked at IBR with ADM with and without radiotherapy in n=91 patients; this showed no significant difference in the revision surgeries in the 2 group. Still, the rate of capsular contracture was higher in the RT group^37^.
2020	*Leonardo Cattelani,* *Susanna Polotto*	One-step PPBR with porcine ADM followed by PMRT is well tolerated with no significant risk of adverse outcomes, in the short-term follow-up^38^.

PMRT- Postmastectomy radiotherapy, RT- Radiotherapy, ADM- Acellular Dermal Matrix, PPBR- Prepectoral Breast Reconstruction.

**Table 3:** Complication range rates in two groups

**Complications (Range %)**	**Full Wrap**	**Partial Wrap**
Implant Loss Rate (%)	3-3.5 (In Mesh) 1-18 (In ADM)	0-8 (In ADM) N/R (In Mesh)
Capsular Contracture Rate (%)	<4 (In Mesh) 2-10 (In ADM)	0-2 (In ADM) N/R (In Mesh)
Seroma Rate (%)	0-14 (In Mesh) 2-24 (In ADM)	0-13 (In ADM) N/R (In Mesh)
Cosmesis/ Rippling (%)	3-4 (In Mesh) 1-36 (In ADM)	12-13 (In ADM) N/R (In Mesh)
Surgical Site Infection rate (%)	0-5 (In Mesh) 1-10 (In ADM)	4-43 (In ADM) N/R (In Mesh)

N/R= Not Recorded

## CONCLUSION

As the demand for breast reconstruction increases newer and novel ways of reconstruction have been used to provide both cosmesis and oncological safety, florid use of subcutaneous mastectomy, the role of Prepectoral Implant-based reconstruction has gained even more popularity. The Prepectoral breast reconstruction with an implant following mastectomy using synthetic mesh or Acellular Dermal Matrix(ADM) provides an adequate soft tissue coverage to the implant under the skin with near comparable outcomes to subpectoral implant-based reconstruction. With this review, we believe, the use of Acellular Dermal Matrices/ Synthetic Mesh with Prepectoral breast reconstruction is both an efficient and effective mode of breast reconstruction, causing minimal morbidity whilst providing good cosmesis.

## FINANCIAL DISCLOSURES

None

## CONFLICT OF INTEREST

The authors declare that there is no conflict of interests.
